# Research Progress on the Molecular Mechanism of LRP1 and TGFβ-PDGFRβ Signaling Network in Atherosclerosis and Vascular Remodeling

**DOI:** 10.3390/ijms27125421

**Published:** 2026-06-16

**Authors:** Xuan Guo, Shuang Xue, Qiao Wang, Xingtong Chen, Jinbiao Yang, Yunyue Zhou, Yukun Zhang, Wenying Niu

**Affiliations:** School of Basic Medical Sciences, Heilongjiang University of Chinese Medicine, Harbin 150040, China

**Keywords:** LRP1, TGFβ, PDGFRβ, atherosclerosis, vascular remodeling

## Abstract

Atherosclerosis (AS) is the primary underlying cause of cardiovascular and cerebrovascular diseases. The occurrence and development of AS are closely related to lipid deposition, chronic inflammation, phenotypic modulation of vascular smooth muscle cells (VSMCs), and extracellular matrix (ECM) remodeling. Numerous studies indicate that low-density lipoprotein receptor-associated protein 1 (LRP1), as a multifunctional receptor, contributes to vascular homeostasis in AS and vascular remodeling by regulating lipid handling, inflammatory responses, transforming growth factor beta (TGFβ) signaling, and platelet-derived growth factor receptor beta (PDGFRβ) trafficking. Rather than treating the LRP1-TGFβ-PDGFRβ relationship as a fully established linear pathway, this review distinguishes demonstrated mechanisms from inferred cross-talk and proposes an integrated, cell- and stage-dependent regulatory model. This article systematically elaborates on the structure and function of LRP1; LRP1-mediated regulation of TGFβ and PDGFRβ in AS and vascular remodeling; the possible relationship among LRP1, TGFβ, and PDGFRβ; and cell-specific effects in VSMCs, macrophages, endothelial cells, and pericytes. Meanwhile, this article summarizes potential translational strategies such as lipid-lowering, anti-inflammatory therapy, PDGFRβ inhibitor repositioning, TGFβ pathway modulation, biomarker-based stratification, and LRP1-targeted delivery. A deeper understanding of the cell-specificity and stage-dependence of the LRP1-TGFβ-PDGFRβ signaling network may help elucidate the progression mechanism of AS and provide new ideas for risk stratification and precise intervention.

## 1. Introduction

Atherosclerosis (AS) is a chronic progressive vascular disease characterized by lipid deposition, inflammatory cell infiltration, and plaque formation in the arterial wall; it is the pathological basis of myocardial infarction, stroke, and peripheral vascular disease. Vascular remodeling is closely intertwined with AS: atherosclerotic lesions drive adaptive or pathological changes in vascular wall structure, whereas remodeling of the vessel wall can influence local hemodynamics, lesion growth, plaque composition, and clinical complications. Therefore, the relationship between AS and vascular remodeling should be viewed as reciprocal and context-dependent rather than a simple one-way causal process. These changes involve phenotypic modulation of vascular smooth muscle cells (VSMCs), extracellular matrix (ECM) remodeling, endothelial dysfunction, and inflammatory cell infiltration [1]. To maintain vascular homeostasis, multiple signaling pathways and membrane receptors work synergistically. Low-density lipoprotein receptor-associated protein 1 (LRP1), as a multifunctional receptor, integrates lipid metabolism, signal transduction, endocytosis, and vascular protection [2]. It may form a regulatory network with transforming growth factor beta (TGFβ) and platelet-derived growth factor receptor beta (PDGFRβ) signaling, thereby influencing the occurrence and progression of atherosclerosis and vascular remodeling [3,4].

## 2. Research Progress of LRP1 and Its Relationship with TGFβ-PDGFRβ Signaling Network

### 2.1. Research Progress of LRP1

#### 2.1.1. The Structure and Function of LRP1

Oxidized low-density lipoprotein (oxLDL) is generated when LDL particles enter the subendothelial intima through a dysfunctional endothelial barrier, bind to extracellular matrix components such as proteoglycans, and undergo oxidative modification mediated by reactive oxygen species, lipoxygenases, myeloperoxidase, and other local pro-oxidant systems. Thus, oxLDL is not simply LDL-C in the blood that is directly oxidized after “flowing into” the intima; rather, retention of LDL in the arterial wall and local oxidative stress jointly promote LDL modification. OxLDL can initiate early AS lesions and amplify lesion progression by activating endothelial cells, recruiting monocytes, inducing macrophage foam-cell formation, and stimulating inflammatory signaling [5]. During this process, low-density lipoprotein receptor-associated protein 1 (LRP1) plays an important regulatory role. LRP1 belongs to the low-density lipoprotein (LDL) receptor family and is a multifunctional transmembrane receptor protein widely expressed on vascular-associated cells. Its unique structure and function enable LRP1 to participate in lipid metabolism, inflammation regulation, efferocytosis, and signal transduction, and it is closely related to the occurrence and development of atherosclerosis [6].

**Figure 1:**
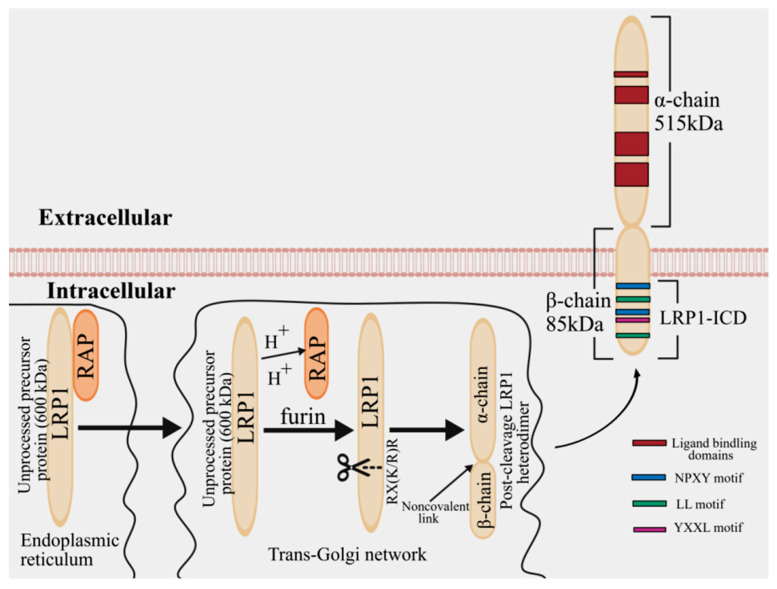
The maturation process of the LRP1 transmembrane receptor. Created with BioGDP.com [9]. Red blocks represent ligand-binding domains; blue indicates the NPXY motif, green denotes the LL motif, and purple marks the YXXL motif.

LRP1 is first synthesized in the form of a single-chain precursor protein with a molecular weight of approximately 600 kDa. The precursor protein does not possess the complete biological function of a mature receptor and only exists in the endoplasmic reticulum or Golgi apparatus. Under the action of Flynn protease, it is cleaved into a 515 kDa alpha chain and an 85 kDa beta chain. The alpha and beta chains form a stable heterodimer structure through noncovalent bonding, ultimately becoming a functional transmembrane receptor protein anchored on the cell membrane surface, namely the LRP1 mature receptor, as shown in Figure 1. The ligand-binding function of the alpha chain is the initial step for LRP1 to exert its biological effects, while the beta chain participates in anchoring and signal transduction. The alpha chain is completely located outside the cell and has four different ligand-binding repeat sequence clusters, which are crucial for interacting with various ligands such as proteases, growth factors, and ECM proteins. These binding clusters contain homologous domains of epidermal growth factor (EGF) precursor and six YWTD repeat sequences, forming a β-helix structure that enhances the specificity of ligand interactions [7]. The beta chain is a subunit that runs through the cell membrane, consisting of a transmembrane domain and an intracellular domain (LRP1-ICD). The transmembrane domain is composed of hydrophobic amino acids embedded in the lipid bilayer of the cell membrane, anchoring the entire LRP1 receptor to the cell membrane and connecting the extracellular alpha chain with the intracellular domain. The intracellular domain contains multiple amino acid sequences, such as the NPXY motif and YXXL motif. These amino acid sequences contain multiple phosphorylation sites and protein binding sites, and can independently complete signal transduction without relying on alpha chains. When the alpha chain binds to the ligand, it activates the intracellular domain through conformational changes, recruiting downstream signaling molecules and initiating intracellular signaling pathways to regulate physiological processes such as phagocytosis, migration, and lipid metabolism [8].

#### 2.1.2. LRP1 Is the Core Regulatory Hub of Lipid Metabolism and Vascular Homeostasis in AS

In AS, when the α chain binds to ligands, the LRP1-ICD of the β chain is activated, which reduces the recruitment of inflammatory cells and the secretion of inflammatory factors such as TNF-α and IL-6 by regulating the NF-κB pathway, alleviating vascular intimal inflammation damage. The β chain regulates the proliferation and migration of VSMCs through the PI3K/Akt pathway, maintaining the stability of the fibrous cap [10,11]. In addition, the alpha chain and beta chain also mediate macrophage phagocytosis and clearance of apoptotic cells, reducing the expansion of necrotic cores and ensuring the stability of plaques [12,13].

##### Regulatory Role of LRP1 in Lipid Metabolism

LRP1 can combine and degrade a variety of atherogenic lipoproteins such as ox LDL, coordinate to maintain the normal concentration of various lipoproteins in the blood, and activate the reverse cholesterol transport pathway, so that the excess cholesterol in the cell is transported to high-density lipoprotein (HDL), and then back to the liver, so that cholesterol is metabolized and discharged, reducing the accumulation of lipid in the cell, and inhibiting the formation of foam cells [14].

##### Regulatory Role of LRP1 in Vascular Biology

In vascular biology, LRP1 promotes the proliferation and migration of vascular smooth muscle cells and the synthesis of collagen matrix, thickens the fibrous cap of atherosclerotic plaque, and inhibits the abnormal apoptosis of vascular smooth muscle cells; LRP1 protects the endothelium from oxidative stress and inflammatory damage, maintains endothelial barrier function, and blocks lesion initiation [15]; LRP1 directly binds to inflammatory mediators such as tumor necrosis factor alpha (TNF-α) and interleukin (IL), blocking the activation of inflammatory signaling pathways, while downregulating the expression of endothelial cell adhesion molecules, inhibiting the recruitment of monocytes to the vascular endothelium, and delaying the progression of atherosclerosis.

In addition to the regulatory effects mentioned above, LRP1 also has significant cell specificity in vascular biology, and the co-receptor activity of LRP1 is regulated by cell type and microenvironment. Fluctuations in oxidative stress levels and changes in the concentration of inflammatory factors can regulate the activity status of LRP1 through signal transduction, thereby affecting the functional output of LRP1 [16]. When LRP1 expression is deficient or functionally impaired in vascular wall cells, a series of pathological reactions are triggered, with the first being the destruction of the integrity of the basement membrane, manifested as reduced collagen IV synthesis, loose basement membrane structure, and more serious pathological conditions such as blood–brain barrier dysfunction [17]. LRP1 also plays a crucial role in regulating the stability of AS plaques, influencing the development of plaques by modulating the functional status and phagocytosis of macrophages. When LRP1 on the surface of macrophages is activated, the cells transform into an anti-inflammatory M2 phenotype, promoting the secretion of immunosuppressive cytokine CXCL1, but relying on downstream signaling pathways to provide an anti-inflammatory microenvironment for plaque stability [18,19,20,21]. In addition, M1/M2 polarization imbalance of macrophages is an important factor in inducing plaque instability, while LRP1-induced M2 phenotype can correct this imbalance and further consolidate plaque stability [22,23].

##### Cross-Regulatory Role of LRP1 in Lipid Metabolism and Vascular Biology

In addition, LRP1 is also a key molecule for achieving cross-regulation of lipid metabolism and vascular biology. In terms of core regulatory mechanisms, LRP1 exerts a dual regulatory effect by regulating the polarization state of macrophages: firstly, LRP1 inhibits macrophage polarization towards pro-inflammatory M1, promotes macrophage transformation towards anti-inflammatory M2, reduces the secretion of pro-inflammatory factors, enhances lipid clearance ability, and achieves dual regulation of vascular inflammation and lipid metabolism. The second is that LRP1 inhibits the expression and signaling of inflammatory factors, reduces the inducible upregulation of CD47, forms a positive cycle of “inhibiting inflammation → reducing CD47 → enhancing cell burial”, effectively blocks the inflammatory cascade reaction within plaques, and reduces the necrotic core volume [24,25]. In addition, in the dual regulation of lipid metabolism and vascular homeostasis, LRP1 precisely regulates endocytosis to mediate lipid ligand clearance and apoptotic cell phagocytosis, while regulating the activity of signaling pathways such as PAI-1, PDGF, and TGFβ to synergistically maintain the structural integrity and functional stability of blood vessels, and maintain vascular microenvironment homeostasis [6].

### 2.2. Research Progress on the Regulatory Effect of LRP1 on TGFβ

#### 2.2.1. Biological Characteristics of TGFβ

Transforming growth factor beta (TGFβ) is a multifunctional cytokine superfamily member widely present in multicellular organisms, including multiple subtypes such as TGFβ1, TGFβ2, and TGFβ3. TGFβ1 plays the main role in the vascular system. TGFβ is synthesized and secreted in its inactive precursor form (latent TGFβ), and activated through protease hydrolysis, integrin-mediated conformational changes, etc., becoming a biologically active homodimer that binds to cell-surface receptors to initiate signal transduction. As a multifunctional regulatory factor, TGFβ participates in various physiological processes such as embryonic development, tissue repair, and immune homeostasis regulation. In the vascular system, it regulates the proliferation, differentiation, apoptosis, and extracellular matrix synthesis of vascular cells, becoming a core factor in vascular remodeling and maintaining the homeostasis of atherosclerotic plaques.

#### 2.2.2. Signal Regulatory Mechanism of LRP1 as a Non-Classical Co-Receptor of TGFβ

**Figure 2:**
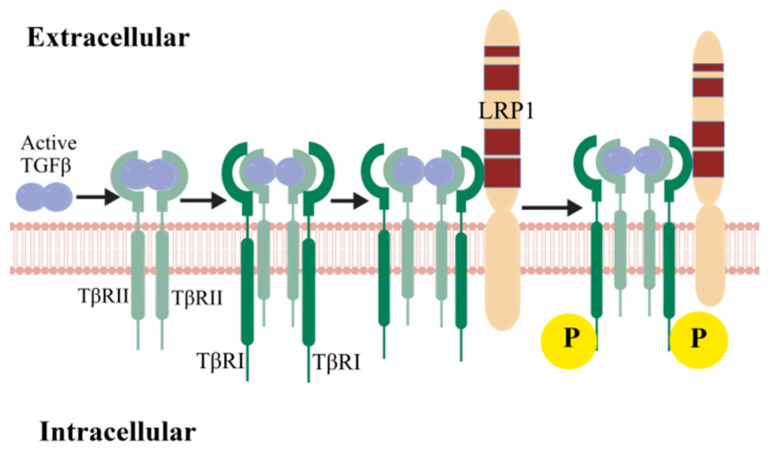
The process of forming a complex between LRP1 and TGFβ. Created with BioGDP.com [9]. Light purple spheres represent active TGFβ ligands; light green structures denote TβRII receptors; dark green structures denote TβRI receptors; beige shapes represent the LRP1 protein; yellow circular marks indicate phosphorylation sites.

**Figure 3:**
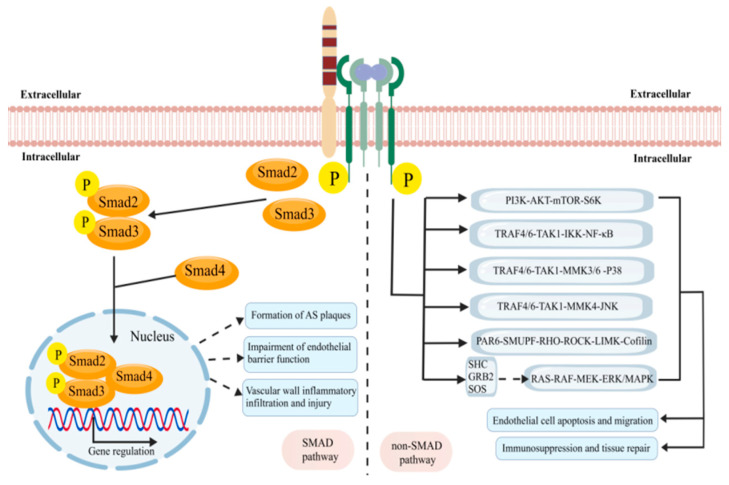
Schematic diagram of LRP1-related TGFβ signaling in Smad and non-Smad pathways regulating AS. Created with BioGDP.com [9]. Beige structure represents LRP1; green shapes denote TβR receptors; orange ovals indicate Smad family proteins; yellow circular marks stand for phosphorylation sites; light blue boxes list downstream non-Smad signaling cascades.

Co-receptors are auxiliary proteins that can regulate the intensity and duration of main receptor signals by enhancing ligand receptor binding, stabilizing receptor complexes, and recruiting downstream signaling molecules [26]. LRP1, as a V-type receptor (TβR-V) of TGFβ, is a co-receptor molecule of TGFβ. It can bind to TGFβ1 and synergistically assemble receptor complexes with TβRI/TβRII. Through non-classical co-receptor mechanisms, it participates in the regulation of TGFβ signaling and enhances downstream biological effects such as growth inhibition and fibrosis. Under physiological conditions, active TGFβ homodimers first bind to high-affinity TβRII to form a TGFβ-TβRII binary complex, and then recruit TβRI to form a TGFβ-TβRII-TβRI ternary complex. At the same time, LRP1 can directly bind to free or receptor-bound TGFβ ligands (biologically active TGFβ protein molecules, i.e., TGFβ1 homodimers) through specific structural sites in the extracellular domain of LRP1, or specifically bind to ternary receptor complexes, ultimately forming a stable multi-component complex, increasing the stability of ligand receptor binding and the membrane localization efficiency of receptor complexes, reducing complex dissociation, as shown in Figure 2. In this complex, the receptor TβRII with intrinsic kinase activity specifically phosphorylates the GS domain (glycine serine enriched region) of the intracellular segment of TβRI, causing conformational changes and complete activation of TβRI kinase, thereby activating the classical Smad2/3/4 pathway and non-Smad pathway, regulating the transcription of downstream target genes, and mediating various physiological processes, as shown in Figure 3 [27].

#### 2.2.3. The Regulatory Role of LRP1-TGFβ Pathway in AS Plaques

During the formation and development of AS plaques, the regulatory effect of TGFβ should be understood as context-dependent rather than simply protective in early lesions and harmful in late lesions. LRP1, as a regulatory node of the TGFβ signaling pathway, may influence plaque biology through cell-specific mechanisms and through differential activation of canonical Smad and non-Smad pathways. In relatively stable or reparative contexts, TGFβ/Smad2/3 signaling can restrain excessive inflammatory activation, support extracellular matrix production, and contribute to fibrous cap formation. However, the same pathway may produce different outcomes when receptor composition, local inflammatory status, mechanical stress, lesion stage, and cell type change. Therefore, the following model is presented as a synthesis of available evidence and plausible cross-talk rather than as a fully demonstrated linear mechanism [3,4].

In VSMCs, persistent TGFβ activation may promote ECM deposition, fibrosis, and calcification, whereas loss or impairment of LRP1 can alter Smad2/3 signaling, increase TGFβ target genes such as thrombospondin 1 (TSP1) and PDGFRβ, and aggravate elastic lamina disruption and vascular fibrosis [28,29,30]. In macrophages, altered LRP1 expression can affect inflammatory polarization, efferocytosis, and TGFβ-related anti-inflammatory signaling, thereby influencing necrotic-core expansion and plaque stability. In endothelial cells, dysregulated TGFβ signaling may contribute to endothelial barrier dysfunction and endothelial–mesenchymal transition. These observations support a cell- and stage-dependent model in which LRP1 modifies TGFβ signaling output but does not act as a universal determinant of a single biological outcome [31,32,33].

#### 2.2.4. Regulatory Role of the LRP1-TGFβ Pathway in Endothelial Barrier Function

LRP1 regulates the TGFβ signaling pathway, forms an LRP1-TGFβ regulatory pathway, and participates in the regulation of endothelial barrier function. Under normal physiological conditions, LRP1 upregulates the expression of endothelial nitric oxide synthase (eNOS) by mediating TGFβ signaling, promoting the synthesis and localization of tight junction proteins, enhancing the connectivity between endothelial cells, and maintaining the integrity of the endothelial barrier [34]. In addition, LRP1 can prevent endothelial–mesenchymal transition (EndMT) caused by its abnormal accumulation by clearing excessive extracellular TGFβ, further maintaining the stability of endothelial cell phenotype [35]. In the pathological microenvironment, the downregulation of LRP1 expression leads to an imbalance in TGFβ signaling regulation. On the one hand, it can reduce the synthesis of tight junction proteins by inhibiting Smad2/3 phosphorylation. On the other hand, it can induce the activation of non-Smad pathways, promote endothelial cell apoptosis and migration, exacerbate endothelial barrier damage, increase vascular permeability, and trigger the occurrence of vascular homeostasis imbalances such as plasma component extravasation and inflammatory cell infiltration [36]. In addition, extracellular vesicles derived from endothelial cells under disturbed blood flow can promote monocyte aggregation and macrophage inflammatory polarization, further accelerating the progression of atherosclerosis [37].

#### 2.2.5. Regulatory Role of LRP1-TGFβ Pathway in Macrophages

In maintaining vascular homeostasis, LRP1 enhances the anti-inflammatory effect of macrophages by regulating the activity of the TGFβ signaling pathway. Under normal physiological conditions, LRP1-mediated TGFβ signaling is activated by inhibiting the NF-κB pathway in macrophages and downregulating the expression of pro-inflammatory factors such as TNF-α, IL-6, and IL-1β, while promoting the secretion of anti-inflammatory factors IL-10 and TGFβ themselves [38], maintaining the M2 anti-inflammatory phenotype of macrophages, and inhibiting the occurrence of local chronic inflammation in blood vessels. In addition, LRP1 creates a favorable microenvironment for TGFβ anti-inflammatory signaling by clearing excess inflammatory mediators and TGFβ inhibitors around macrophages [15]. Under pathological conditions, the expression of LRP1 in macrophages decreases, leading to insufficient activation of the TGFβ signal, breaking the proinflammatory/anti-inflammatory balance, inducing macrophages to transform into M1 proinflammatory phenotype, thus intensifying inflammatory infiltration and damage of vascular walls, destroying vascular homeostasis, and promoting the occurrence and development of vascular diseases such as atherosclerosis [39].

### 2.3. Research Progress on the Regulatory Effect of LRP1 on PDGFRβ

#### 2.3.1. Biological Characteristics of PDGFRβ

Platelet-derived growth factor receptors (PDGFRs) are members of the III receptor tyrosine kinase family widely expressed on the cell surface, mainly consisting of two subtypes: PDGFRα and PDGFRβ. PDGFRβ plays the most important functional role in vascular wall-associated cells. PDGFRβ is synthesized in an inactive monomeric form and localized on the cell membrane surface. It forms homodimers by binding with platelet-derived growth factor (PDGF) ligands (mainly PDGF-BB and PDGF-AB subtypes) or heterodimers by binding with PDGFRα, leading to phosphorylation of the intracellular tyrosine kinase domain and becoming a receptor complex with signaling activity. PDGFRβ is a type of proliferative signaling receptor that can participate in various physiological processes such as embryonic vascular development, repair of tissue damage, and maintenance of vascular homeostasis. PDGFRβ becomes an important molecule regulating vascular remodeling and AS plaque development by regulating the proliferation, migration, and extracellular matrix synthesis of vascular wall cells such as vascular smooth muscle cells and pericytes [40].

#### 2.3.2. The Endocytic Degradation Mechanism of PDGFRβ Mediated by LRP1

**Figure 4:**
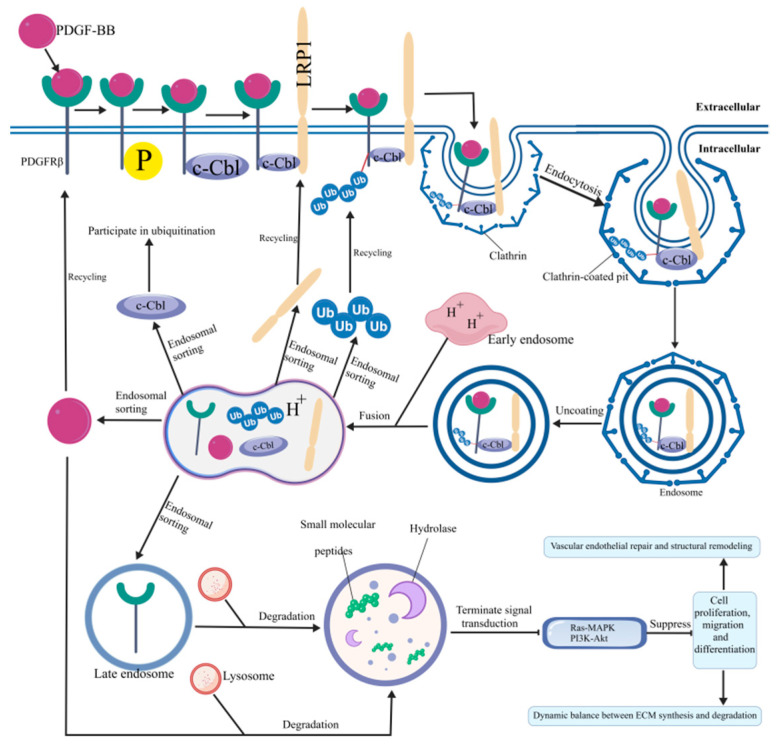
LRP1-mediated endocytosis and degradation of PDGFRβ and its consequences for cell proliferation, migration, and differentiation. Created with BioGDP.com [9]. Magenta spheres represent PDGF-BB ligand; light green structures indicate PDGFRβ receptors; beige elongated shapes denote LRP1 protein; yellow circular marks represent phosphorylation sites; light blue layered vesicles denote clathrin-coated pit, early endosome, late endosome compartments; grey ovals represent c-Cbl ubiquitin ligase; blue Ub tags stand for ubiquitin molecules; light pink circular organelle represents lysosome; pale purple oval inside lysosome marks hydrolase enzyme; light blue rectangular boxes denote downstream signaling pathways and biological functional outcomes; black directional arrows illustrate endocytic trafficking, recycling and protein degradation flow.

LRP1, as a multifunctional endocytic signaling receptor, exhibits a highly expressed characteristic form in VSMC and is a negative regulator of the platelet-derived growth factor BB (PDGF-BB) signaling pathway. After binding to PDGF-BB and PDGFRβ, downstream proliferation signals are activated, but excessive activation can lead to abnormal proliferation of VSMCs and disrupt vascular homeostasis. LRP1 can limit the sustained activation of the signaling pathway by mediating the endocytic degradation of PDGFRβ, as shown in Figure 4. Firstly, PDGFRβ binds to PDGF-BB on the surface of VSMCs, inducing PDGFRβ dimerization and self-phosphorylation, resulting in activated PDGFRβ. Then, activated PDGFRβ recruits ubiquitin ligase c-Cbl through phosphorylation sites, and recruits LRP1 through c-Cbl bridging or direct binding to form the LRP1-PDGFRβ-c-Cbl ternary complex. C-Cbl acts as an E3 ubiquitin ligase, connecting ubiquitin molecules to the intracellular lysine residues of PDGFRβ, promoting c-Cbl-mediated K63 polyubiquitination of PDGFRβ. This ubiquitination tag serves as the initiating signal for endocytosis. The clathrin on the cell membrane recognizes the ubiquitinated tag of PDGFRβ and assembles around PDGFRβ to form clathrin-coated caveolas. Dynamin mediates the detachment of the coated caveolas from the cell membrane, forming clathrin-coated bodies (i.e., endosomes) that transport PDGFRβ from the cell membrane to the cytoplasm. After removing the clathrin shell, the endosomes fuse with the early endosomes. After sorting, the endosomes carrying PDGFRβ fuse with lysosomes. The acidic hydrolytic enzymes in the lysosome degrade PDGFRβ into small peptide segments, clearing the active PDGFRβ on the cell surface and terminating downstream proliferation signaling pathways mediated by PDGF-BB, such as the Ras MAPK pathway and PI3K Akt pathway [41,42].

The disruption of endocytic circulation mediated by LRP1 can lead to abnormal accumulation of PDGFRβ on the cell membrane surface, persistent overactivation of downstream signaling pathways, abnormal proliferation of VSMCs, pathological changes such as vascular wall structural remodeling and reduced elasticity, ultimately exacerbating vascular homeostasis imbalance. In the pathological state of thymidine β4 (Tβ4) deficiency, VSMCs exhibit increased sensitivity to PDGF-BB, manifested by an abnormally high recycling efficiency of LRP1-PDGFRβ complex and obstruction of lysosomal transport and degradation. This manifestation may be directly related to the dysregulation of LRP1-regulated endocytosis [43]. However, the intervention of Imatinib, an inhibitor targeting PDGFRβ, can effectively reverse the exacerbated aneurysm phenotype in Tβ4-deficient mouse models, indicating that the LRP1-dependent PDGFRβ endocytic degradation regulatory mechanism plays a core role in maintaining vascular structural integrity and functional stability [41,42,44].

#### 2.3.3. Regulatory Role of the LRP1-PDGFRβ Pathway in Vascular Remodeling and Plaque Biology

In carotid artery ligation or vascular injury models, VSMC-specific LRP1-deficient mice exhibit significantly thickened neointima, excessive collagen deposition, and elastic fiber rupture, confirming that LRP1 maintains vascular structural homeostasis partly by limiting PDGFRβ signaling. In the early stages of vascular injury, moderate PDGFRβ activation can promote VSMC repair responses, including proliferation, migration, and matrix production. However, in atherosclerotic plaques, VSMC biology extends beyond a simple contractile-to-synthetic switch. Lineage-tracing and single-cell studies show that VSMCs can transition toward macrophage-like, osteogenic-like, fibrochondrocyte-like, fibromyocyte, and foam-cell-like states and can contribute substantially to the fibrous cap and plaque cellular composition [40,45,46,47,48,49,50]. Therefore, persistent PDGFRβ activation should be interpreted not only as a driver of proliferation and migration but also as a signal that may influence the spectrum of modulated SMC states, ECM remodeling, fibrous-cap formation, and plaque stability [51,52].

#### 2.3.4. The Regulatory Role of the LRP1-PDGFRβ Pathway in ECM Metabolism

The basis for maintaining vascular wall structure is the dynamic balance between ECM synthesis, cross-linking, and degradation. The LRP1-PDGFRβ pathway contributes to this balance by regulating VSMC state, matrix turnover, and protease activity. PDGFRβ activation can promote MMP2/9 and LOX-related remodeling, whereas LRP1-mediated endocytosis and degradation of PDGFRβ may limit excessive downstream signaling and help maintain ECM homeostasis. In plaques, disturbed LRP1-PDGFRβ signaling may therefore influence fibrous-cap thickness, matrix organization, and vulnerability rather than merely increasing or decreasing a single ECM component [53,54].

In human AS plaque tissue, the down-regulation region of LRP1 expression is often accompanied by the high expression of PDGFRβ protein and the significant increase in MMP2/9 activity. This phenomenon is directly related to the increased degradation of the ECM and thinning of the fibrous cap in the plaque, thereby increasing plaque vulnerability and risk of rupture. Clinical pathological studies have confirmed that the expression level of LRP1 is positively correlated with the stability of AS plaques [38], and its loss of expression can disrupt ECM homeostasis through excessive activation of PDGFRβ signaling, accelerating the progression of AS.

#### 2.3.5. PDGFRβ Signaling in Plaque Biology and Modern SMC-State Concepts

Beyond receptor trafficking, PDGFRβ signaling is directly relevant to plaque composition and repair. Lineage-tracing and single-cell studies have shown that VSMCs and SMC-derived cells contribute substantially to human and murine lesions, including the fibrous cap and multiple modulated states [34,49,55,56]. These cells are not limited to a contractile-to-synthetic transition; under plaque stimuli, they may acquire macrophage-like, foam-cell-like, osteogenic/chondrogenic, fibromyocyte, inflammatory, or fibroblast activation protein-positive states that influence lipid accumulation, calcification, ECM remodeling, and cap stability [32,33,50,51,52,55,57,58,59,60,61,62,63].

In this context, PDGFRβ should be discussed not only as a proliferative receptor but also as a regulator of SMC survival, migration, bioenergetics, clonal expansion, fibrous-cap formation, and cell–cell communication. Moderate PDGFRβ activity may participate in tissue repair and cap formation, whereas persistent or spatially dysregulated activation may promote intimal thickening, matrix imbalance, and plaque progression. Recent spatial and single-cell transcriptomic studies further indicate that plaque microdomains contain distinct SMC, macrophage, endothelial, and immune-cell neighborhoods, making the effect of PDGFRβ highly dependent on local cellular context [3,4,47,56,64,65,66,67].

### 2.4. Dual Directional Regulatory Effect of TGFβ on PDGFRβ

**Figure 5:**
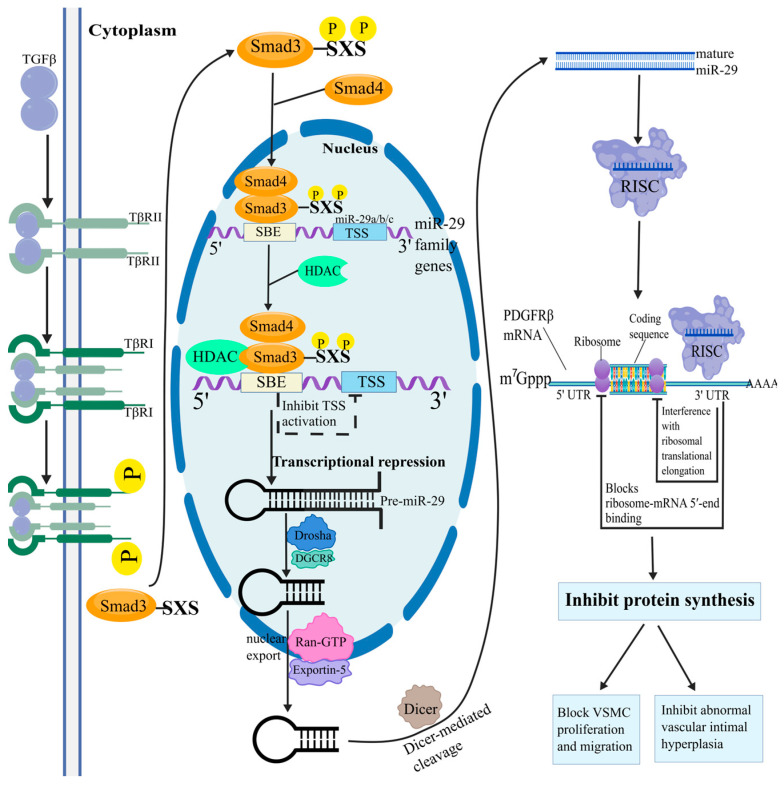
Mechanism by which TGFβ/Smad signaling may inhibit PDGFRβ protein synthesis through transcriptional and miR-29-associated post-transcriptional mechanisms. Created with BioGDP.com [9]. Light purple spheres: mature active TGFβ ligand; light green membrane structures: TβRII transmembrane receptors; dark green membrane structures: TβRI transmembrane receptors; orange oval shapes: Smad3 and Smad4 transcription factor proteins; yellow solid circular marks: phosphorylation modification sites; rounded rectangular/oval structures: nuclear regulatory proteins including HDAC, Drosha, DGCR8, Ran-GTP, Exportin-5, and cytoplasmic Dicer; black line segments: DNA gene sequences (SBE, TSS, pri-miR-29 coding region); white oval nuclear compartment: cell nucleus; pale beige background area: cytoplasm; light blue complex structure: mature miR-29 bound within RISC complex; m^7^Gppp-labelled linear chain: PDGFRβ mRNA transcript (5′ UTR, coding sequence, 3′ UTR, poly-A tail); grey stacked oval structure: ribosome translational complex; light blue rectangular text boxes: final downstream functional biological outcomes.

TGFβ performs bidirectional regulation of PDGFRβ signaling through the classical Smad-dependent pathway, while LRP1 is not an essential receptor in this process. When involved, it acts as a co-receptor to enhance activation efficiency, and when not involved, the pathway is normally activated. At a physiological steady state, TGFβ binds to the cell membrane type II receptor (TβRII), recruits and activates the type I receptor ALK5 (a subtype of TβRI), phosphorylates the activated ALK5, and further induces phosphorylation of the Smad3 carboxyl terminal SXS motif [68]. Phosphorylation of Smad3 and Smad4 forms a heterologous complex in the nucleus, which achieves negative regulation of PDGFRβ through two synergistic downstream pathways. The first point is that at the level of gene transcription (nuclear), heterologous complexes recruit corepressors such as histone deacetylase (HDAC) by binding to Smad binding elements (SBE) in the promoter region of the PDGFRβ gene, inhibiting the transcription start site (TSS) of the PDGFRβ gene, reducing the mRNA production of PDGFRβ from the source of gene transcription, and downregulating the protein expression of PDGFRβ. The other is that at the mRNA transcription level (cytoplasm), TGFβ regulates the transcriptional expression of the miR-29 family (miR-29a, miR-29b, miR-29c) through the Smad pathway, and different miR-29 members have specific responses to TGFβ stimulation. Mature miR-29 can directly bind to the 3′ non-coding region of PDGFRβ mRNA, blocking translation processes or accelerating mRNA degradation, reducing PDGFRβ protein synthesis at the post-transcriptional level. Primary miR-29 (pri-miR-29) is transcribed by RNA polymerase II or III in the nucleus. The endonuclease Drosha forms a complex with the double-stranded RNA-binding protein DGCR8 and acts on pri-miR-29, causing it to be spliced and resulting in a stem circular pre-miR-29 approximately 70 nt in length with a 3′ end protrusion. Under the coordination of Ran GTP, Exportin-5 transported pre-miR-29 from the nucleus to the cytoplasm. In the cytoplasm, pre-miR-29 is cleaved into mature double-stranded miR-29 of 22 bp by the Dicer enzyme. Mature miR-29 is recruited into the RNA-induced silencing complex (RISC) and binds to the 3′UTR of PDGFRβ mRNA. It directly inhibits the synthesis of PDGFRβ protein by blocking the binding of ribosomes to the 5′ end of mRNA or interfering with the translation extension process of ribosomes, but does not cause mRNA degradation. At this time, there is no significant change in the mRNA level of PDGFRβ in cells, while the protein level is significantly reduced. When miR-29 is fully complementary to the 3′UTR of PDGFRβ mRNA, it recruits nucleases (such as Ago2 endonuclease activity) to cleave the mRNA chain or promote mRNA deacetylation (removal of poly (A) tails), which is then cleared by intracellular degradation pathways (such as exosome complexes), accelerating mRNA degradation and leading to a decrease in PDGFRβ mRNA levels and protein synthesis, as shown in Figure 5 [69].

These two pathways form a dual inhibitory effect at the levels of “transcription initiation” and “post transcriptional processing”, which results in the inability of PDGFRβ gene to synthesize protein smoothly even after transcription into mRNA, significantly reducing the protein expression of PDGFRβ and directly inhibiting the excessive proliferation and phenotype transformation of VSMCs, while promoting the expression of α-SMA, collagen, etc., enhancing vascular wall elasticity, and maintaining vascular wall structural integrity. However, in pathological conditions, Smad3 may release the inhibition of PDGFRβ through mechanisms such as mediating p27 nuclear output, while the ALK1 (a subtype of TβRI) pathway dominates and promotes PDGFRβ-mediated VSMC proliferation through Smad1/5/8, participating in vascular endothelial proliferation and remodeling. In pathological environments, TGFβ regulates non-classical signaling pathways by activating the mitogen-activated protein kinase (MAPK) pathway, synergistically enhancing VSMC migration with PDGF-BB, and accelerating the process of vascular pathological remodeling. TGFβ can also phosphorylate and activate the PDGFRβ catalytic domain, initiating the Akt/PRAS40 signaling pathway to exacerbate abnormal cellular activation. At this point, LRP1 exerts a bidirectional regulatory effect by maintaining the activation state of TGFβ signaling, inhibiting Smad3-mediated PDGFRβ expression under physiological conditions, and blocking the excessive activation of non-classical TGFβ pathways and abnormal phosphorylation of PDGFRβ in pathological environments.

### 2.5. LRP1 and TGFβ-PDGFRβ Signaling Network Regulatory Mechanism

**Figure 6:**
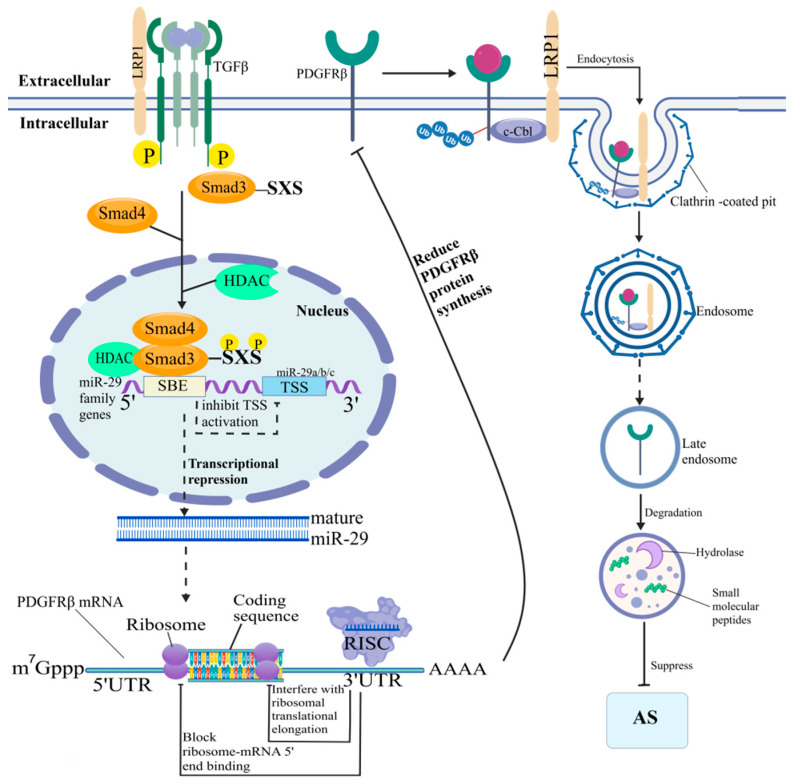
Conceptual model of the LRP1-TGFβ-PDGFRβ signaling network. Created with BioGDP.com [9]. Beige elongated transmembrane structures: LRP1 receptor protein; light green bilayer membrane structures: TβRII receptors; dark green bilayer membrane structures: TβRI receptors; single light green curved membrane structure: PDGFRβ receptor; magenta circular sphere: PDGF-BB ligand; yellow solid circular marks: protein phosphorylation modification sites; orange oval shapes: Smad3 and Smad4 transcription factor proteins; light green rounded rectangular structures: HDAC epigenetic regulatory protein; blue small circular tags: ubiquitin (Ub) molecules; grey oval shape: c-Cbl ubiquitin ligase; purple-blue layered hollow vesicles: clathrin-coated pit, early endosome, late endosome, intracellular compartments; light purple oval inside late endosome: hydrolase degrading enzyme; purple dashed rounded compartment: cell nucleus; pale off-white background region: cytoplasm; blue horizontal linear strip: mature miR-29 RNA transcript; m^7^Gppp-labelled linear nucleic acid chain: PDGFRβ mRNA, containing 5′ UTR, coding sequence, 3′ UTR and poly-A tail; grey stacked oval complexes: ribosome translational machinery; dark purple irregular cloud-shaped complex: RISC miRNA-effector complex; light blue rectangular box: atherosclerotic lesion (AS) pathological phenotype; black solid and dashed directional arrows: signal transduction, endocytosis, and transcriptional/translational inhibitory flow.

LRP1 is a key regulatory molecule in the proposed LRP1-TGFβ-PDGFRβ signaling network. LRP1 independently regulates aspects of TGFβ and PDGFRβ signaling and may mediate cross-pathway dialogue between them. However, direct evidence for a single integrated pathway containing all three components remains incomplete; therefore, the network described here should be interpreted as a synthesis of established mechanisms, inferred relationships, and testable hypotheses, as shown in Figure 6.

Under physiological or reparative conditions, LRP1 may help restrain excessive PDGFRβ activation, support efferocytosis, and modulate TGFβ/Smad signaling in a manner that preserves vascular-wall integrity [24,43,70]. Under pathological conditions, reduced LRP1 expression or impaired endocytic function may permit sustained PDGFRβ signaling and altered TGFβ pathway balance, thereby contributing to VSMC phenotypic modulation, inflammatory amplification, ECM remodeling, and plaque vulnerability [2,30,43,71]. This model is plausible but should be interpreted as cell- and stage-specific; direct experimental testing using cell-specific genetic models, lineage tracing, and spatial multi-omics is needed before it can be considered a general mechanism of AS progression [47,64].

### 2.6. Cell-Specific Functions and Evidence Strength of the LRP1-TGFβ-PDGFRβ Signaling Network

A cell-specific framework is essential because LRP1, TGFβ, and PDGFRβ do not produce the same biological output in all vascular cells. In VSMCs, LRP1 is closely linked to PDGFRβ internalization, ubiquitination, and degradation, and LRP1 deficiency can enhance PDGFRβ signaling, Smad2/3 activation, medial degeneration, and neointimal remodeling [43,44,70]. Recent evidence also indicates that VSMCs can generate diverse plaque-cell states, including macrophage-like, foam-cell-like, osteogenic/chondrogenic, fibromyocyte, and FAP-positive modulated cells, which may either stabilize or destabilize plaques depending on location and stage [50,57,58].

In macrophages, LRP1 is particularly important for efferocytosis and inflammatory resolution. Macrophage LRP1 is required for the anti-CD47 strategy to enhance efferocytosis, reduce lesion size, and decrease necrotic-core formation in Apoe^−/−^ models, indicating that macrophage LRP1 affects plaque outcome through mechanisms distinct from VSMC PDGFRβ trafficking [24]. TGFβ in macrophage-rich regions may suppress or promote inflammation depending on macrophage state, lipid burden, and cytokine milieu [28,39,72,73,74,75].

In endothelial cells and pericytes, LRP1 contributes to barrier function, endocytosis, inflammatory signaling, and vascular-wall integrity. Endothelial dysfunction and disturbed-flow-derived extracellular vesicles can reshape macrophage polarization and plaque inflammation, while pericyte- and mural-cell LRP1 may influence microvascular integrity and remodeling [17,34,37,76]. Because TGFβ can also drive EndMT or fibrosis under specific conditions, endothelial and mural-cell responses should not be extrapolated directly from VSMC or macrophage findings [73,77,78,79].

Accordingly, this review grades the evidence as follows: (i) established mechanisms include LRP1-dependent PDGFRβ trafficking in VSMCs, macrophage LRP1-mediated efferocytosis, and context-dependent TGFβ signaling; (ii) inferred relationships include coordinated changes in LRP1, TGFβ/Smad activity, PDGFRβ phosphorylation, ECM remodeling, and VSMC-state transitions within plaques; and (iii) speculative models include a universal LRP1-TGFβ-PDGFRβ network that operates identically across vascular cell types. Future work should combine conditional knockout models, lineage tracing, phosphoproteomics, single-cell RNA sequencing, spatial transcriptomics, and cell–cell communication analysis to test these hypotheses [3,4,47,55,56,64,66,75,80,81,82].

## 3. Clinical Treatment Strategies and Targeted Translation Applications

The treatment of atherosclerosis and vascular remodeling should not rely on inhibition of a single pathological link, because lipid metabolism, inflammation, endothelial dysfunction, VSMC phenotypic modulation, ECM remodeling, and plaque stability interact in a cell- and stage-dependent manner [83,84]. Current clinical therapies mainly reduce LDL-C, control inflammatory risk, and improve vascular function. These therapies do not directly prove the existence of an LRP1-TGFβ-PDGFRβ network, but they can modify upstream conditions that influence LRP1 expression, macrophage efferocytosis, PDGFRβ activity, and TGFβ pathway balance. Therefore, this section explicitly distinguishes clinically established treatments from pathway-directed translational hypotheses.

### 3.1. Clinical Application of Lipid-Lowering and Anti-Inflammatory Therapy in AS

At present, prevention and treatment of atherosclerotic cardiovascular disease remain based on lipid-lowering therapy. Commonly used options include statins, ezetimibe, PCSK9 monoclonal antibodies, inclisiran, and bempedoic acid. The 2024 ESC Chronic Coronary Syndrome Guidelines recommend intensive LDL-C lowering in high-risk patients, and non-statin therapies may be considered when LDL-C targets are not achieved or statins are not tolerated [84]. Mechanistically, lipid-lowering therapy reduces LDL-C and ox-LDL deposition in the vascular intima, decreases macrophage foam-cell formation and inflammatory activation, and may indirectly preserve LRP1-mediated lipid clearance, efferocytosis, and plaque-stabilizing responses [85,86].

In addition to lipid deposition, chronic inflammation is also an important factor driving the progression and rupture of AS plaques [72,87]. Some studies have shown that low-dose colchicine has been used to reduce the risk of atherosclerosis-related cardiovascular events [88]. A total of 0.5 mg colchicine once a day can reduce the risk of myocardial infarction, stroke, coronary artery revascularization, and cardiovascular death in adult patients who have been diagnosed with atherosclerosis [88,89,90]. The 2023 AHA/ACC guidelines for chronic coronary artery disease also include low-dose colchicine as a secondary prevention option [91].

Within the proposed LRP1-TGFβ-PDGFRβ framework, lipid-lowering and anti-inflammatory therapies should be interpreted as upstream modifiers rather than direct network-targeted drugs. Reduced lipid burden may support LRP1-mediated clearance and reduce ox-LDL-driven VSMC foam-cell formation, whereas low-dose colchicine may reduce inflammatory signals that impair efferocytosis and promote plaque vulnerability [14,24,82,87,88,89,90,91]. Potential response biomarkers include LDL-C, hsCRP, LRP1 expression or soluble LRP1, activated TGFβ, phosphorylated Smad2/3, phosphorylated PDGFRβ, MMP2/9 activity, VSMC-state markers, and imaging features of plaque inflammation or cap integrity [2,28,48,49,60,65,66,67,72,74,85,86].

### 3.2. Repositioning the Value of PDGFRβ Inhibitors in Vascular Remodeling

PDGFRβ is an important receptor tyrosine kinase that regulates VSMC proliferation, migration, and phenotype transition [40,57,92]. In the process of atherosclerosis and vascular injury, the continuous activation of the PDGF-BB/PDGFRβ signal can promote the transformation of VSMCs from contractile phenotype to synthetic phenotype, leading to problems such as neointimal thickening, abnormal deposition of extracellular matrix, and vascular wall remodeling [11,57,58]. LRP1 can limit its excessive activation by mediating the endocytosis and degradation of PDGFRβ. Therefore, when LRP1 expression decreases or endocytosis function is impaired, abnormal activation of PDGFRβ may become an important mechanism for promoting vascular remodeling.

These observations suggest that PDGFRβ inhibitors may have drug-repositioning value in selected vascular remodeling contexts rather than as routine anti-atherosclerotic agents. Tyrosine kinase inhibitors such as imatinib can reduce abnormal VSMC proliferation and migration by inhibiting PDGFRβ phosphorylation and downstream PI3K/Akt and ERK/MAPK signaling. Edaravone has also been reported to inhibit PDGF-BB-induced VSMC phenotypic transition and neointimal formation in mice, partly through AKT and ERK1/2 signaling [93]. In addition, OTUB1 regulates VSMC phenotypic transition by affecting PDGFRβ ubiquitination and stability, suggesting that the PDGFRβ phosphorylation–ubiquitination–degradation pathway may represent a more precise intervention direction [71].

Because PDGFRβ also contributes to normal vascular repair, SMC survival, and fibrous-cap formation, broad PDGFRβ inhibition is unlikely to be suitable as routine AS therapy. The more plausible translational niche is precision intervention in settings with demonstrable PDGFRβ overactivation, impaired LRP1-dependent receptor turnover, restenosis, aneurysm-associated remodeling, or high-risk modulated SMC states [40,43,50,71,92]. Druggability may be improved by local delivery, transient dosing, or combination with biomarkers that identify PDGFRβ-dominant lesions.

### 3.3. Clinical Translational Limitations of TGFβ Pathway Targeted Therapy

TGFβ signaling has context-dependent effects in atherosclerosis and vascular remodeling rather than a simple bidirectional role [73,94]. Moderate TGFβ/Smad signaling can suppress inflammation, support VSMC quiescence, and promote fibrous-cap ECM deposition, whereas sustained, mislocalized, or noncanonical TGFβ signaling can promote EndMT, fibrosis, calcification, ECM imbalance, or maladaptive remodeling. Because TGFβ is widely expressed and essential for tissue repair and immune homeostasis, systemic pathway inhibition may cause unacceptable off-target effects [28,31,73,95,96].

The main limitations of clinical translation of the TGFβ pathway are cell specificity and stage dependence. In the early stages of AS, complete inhibition of TGFβ may weaken anti-inflammatory and plaque-stabilizing effects. In the late stage of fibrosis or calcification, moderate inhibition of TGFβ-promoting fibrotic branches may have therapeutic significance [94]. Studies have shown that the inhibition of TGFβ has certain potential in anti-fibrosis, but clinical applications still face challenges in terms of efficacy, safety, and targeting accuracy [95]. Meanwhile, existing TGFβ targeting strategies often struggle to distinguish between different subtypes or disease-specific branches, leading to widespread inhibition rather than precise regulation [27].

In summary, TGFβ-targeted therapy should currently be considered a potential signaling-modulation strategy rather than a mature AS treatment. Future studies should prioritize isoform-specific, branch-specific, and cell-targeted approaches, including local delivery to plaque microdomains and patient stratification based on LRP1 expression, Smad2/3 phosphorylation, PDGFRβ activation, inflammatory markers, and plaque imaging features [48,49,73,94,95].

### 3.4. Drug Delivery and Biomarker Application Based on LRP1

LRP1 has ligand binding, endocytosis transport, and signal regulation functions, so it is not only an important mechanism molecule of atherosclerosis and vascular remodeling, but also a potential target for drug delivery and clinical risk assessment. In recent years, nanomedicine delivery systems have been widely used in the research of AS therapy, with the advantages of improving drug stability, bioavailability, and lesion targeting, as well as reducing systemic off-target effects [97,98]. It has also been reviewed that nano-delivery systems can deliver anti-atherosclerosis drugs to specific cells, microstructures, and molecular targets at the lesion site, thereby improving the poor solubility, insufficient targeting, and obvious adverse reactions of traditional drugs [99].

Based on the characteristics of LRP1-mediated endocytosis, future exploration of LRP1 ligand-modified nanocarriers can be used to deliver anti-inflammatory drugs, lipid-lowering drugs, PDGFRβ inhibitors, miRNA, or gene regulatory molecules to macrophages, endothelial cells, or VSMCs within plaques, increasing local drug concentrations and reducing systemic side effects. Previous studies on LRP1-targeted nano-delivery have shown that LRP1 ligands such as Angiopep-2 can promote LRP1-mediated endothelial cell endocytosis, suggesting that LRP1 as a delivery entry point has certain feasibility. However, this evidence is currently mostly from neurovascular or blood–brain barrier-related models, and further validation is needed for direct use in AS plaque-targeted delivery [100].

In addition, LRP1 also has the potential to serve as a biomarker related to vascular remodeling. The 2024 Circulation Research study showed that LRP1 expression is regulated by SNAIL, and its expression inhibition is associated with extracellular matrix remodeling and genetic risk of vascular diseases, suggesting that changes in LRP1 expression may reflect the stability of vascular wall structure and pathological remodeling status [2]. Therefore, in the future, LRP1 expression levels, soluble LRP1, TGFβ activation fragments, PDGFRβ phosphorylation levels, MMP2/9, and VSMC phenotype markers can be combined to evaluate plaque stability, vascular remodeling degree, and targeted therapy response. These biomarkers may also help determine whether a patient is more likely to benefit from lipid-lowering and anti-inflammatory therapy alone or from future pathway-directed strategies targeting PDGFRβ activation, TGFβ signaling branches, or LRP1-mediated delivery.

In order to further clarify the transformation value of the LRP1-TGFβ-PDGFRβ signal network in the treatment of atherosclerosis and vascular remodeling, this paper summarizes the existing commonly used clinical therapeutic drugs and potential targeted intervention strategies. Different treatment methods do not directly act on LRP1, TGFβ, or PDGFRβ, but can indirectly or directly affect the functional status of this signaling network through lipid regulation, anti-inflammatory effects, inhibition of VSMC abnormal proliferation, improvement of extracellular matrix homeostasis, or enhancement of plaque stability, as shown in Table 1.

## 4. Summary and Future Directions

As a multifunctional receptor with ligand binding, endocytosis, and signal regulation functions, LRP1 plays cell-specific roles in atherosclerosis and vascular remodeling. LRP1 participates in lipid handling, cholesterol metabolism, inflammatory regulation, and efferocytosis, thereby helping maintain the local vascular microenvironment. In VSMCs, LRP1 also regulates PDGFRβ trafficking and interacts with TGFβ-related signaling; in macrophages and endothelial cells, its major outputs include efferocytosis, inflammatory resolution, and barrier protection. Therefore, the LRP1-TGFβ-PDGFRβ network should be understood as a context-dependent framework supported by several established links and additional inferred relationships that require direct testing.

Under physiological conditions, LRP1 helps to maintain the balance between TGFβ-Smad signaling and PDGFRβ signaling, limit abnormal proliferation and migration of VSMCs, and maintain vascular structural stability. In pathological conditions, when the expression of LRP1 decreases or endocytic function is impaired, the imbalance of TGFβ signaling, sustained activation of PDGFRβ, enhanced inflammatory response, and abnormal remodeling of ECM can jointly promote the progression of AS, vascular stenosis, and plaque instability.

At present, research on the LRP1-TGFβ-PDGFRβ signaling network still faces several limitations. First, dynamic monitoring of LRP1 trafficking, PDGFRβ phosphorylation, and Smad/non-Smad pathway activity remains insufficient. Second, LRP1 functions differ among endothelial cells, VSMCs, macrophages, pericytes, and perivascular adipocytes, so conclusions from one cell type cannot be generalized without validation. Third, single-cell RNA sequencing, spatial transcriptomics, lineage tracing, and phosphoproteomics should be integrated to distinguish protective SMC-derived cap cells from macrophage-like, foam-cell-like, osteogenic/chondrogenic, inflammatory, or FAP-positive modulated SMC states [47,50,57,64].

In terms of clinical translation, detecting a single LRP1, TGFβ, or PDGFRβ often makes it difficult to accurately assess the stability of plaques and the degree of vascular remodeling. In the future, a multi-indicator joint evaluation system could be established to combine the expression level of LRP1, soluble LRP1, activated TGFβ, Smad2/3 phosphorylation level, PDGFRβ phosphorylation level, MMP2/9, VSMC phenotype markers, and plaque imaging features to predict plaque vulnerability, evaluate disease progression, and screen populations suitable for targeted interventions [48,49].

Future research should gradually shift from single molecular targets to networked, cell-specific, and precise interventions. On the one hand, it is necessary to further elucidate the synergistic regulatory mechanism of LRP1 on the classical/non-classical pathways of TGFβ and the internalization and degradation process of PDGFRβ. On the other hand, local delivery strategies based on LRP1 can be explored to precisely deliver anti-inflammatory drugs, PDGFRβ inhibitors, miRNAs, or gene regulatory molecules to macrophages, endothelial cells, or VSMCs within plaques, to improve therapeutic targeting and reduce systemic side effects. Meanwhile, the application of PDGFRβ inhibitors and TGFβ pathway modulators in AS should emphasize precise stratification and avoid widespread inhibition. In addition, research on immunotherapy targeting regulatory VSMCs also suggests that lesion cell-specific intervention may become a new direction for precision treatment of AS [50]. In the future, by combining multiple omics data, molecular imaging, machine learning models, and clinical sample validation, it is expected that a risk stratification and precision treatment system will be established based on the LRP1-TGFβ-PDGFRβ signaling network, providing new translational directions for AS and vascular remodeling-related diseases [3,4,33,75,79].

## Figures and Tables

**Table 1:** LRP1-TGFβ-PDGFRβ signaling network-related therapeutic strategies and clinical translational value.

Drug/Treatment Direction	Main Target of Action	The Relationship with the LRP1-TGFβ-PDGFRβ Network	Clinical Application Status
statins	HMG-CoA reductase	Reduce LDL-C and ox LDL load, indirectly improve LRP1-mediated lipid clearance and inflammation regulation.	AS basic treatment
Ezetimibe	NPC1L1	Reduce intestinal cholesterol absorption and assist in reducing lipid deposition.	Commonly used combination lipid-lowering drugs in clinical practice
PCSK9 inhibitor	PCSK9/LDLR pathway	Enhance LDL clearance and reduce plaque lipid core formation	Commonly used in high-risk ASCVD patients
Inclisiran	PCSK9 mRNA	Long-term reduction in LDL-C and improvement of patient compliance	In clinical applications
Bempedoic acid	ATP citrate lyase	Non-statin LDL-C lowering option; reduces lipid load and may indirectly improve LRP1-related lipid clearance and inflammatory tone	Clinical lipid-lowering option, especially for selected statin-intolerant or insufficiently controlled patients
Low-dose colchicine	Inflammasome/inflammatory response	Reducing the inflammatory microenvironment of plaques may indirectly improve LRP1-related steady-state function	Used for ASCVD risk reduction
Imatinib and other PDGFRβ inhibitors	PDGFRβ phosphorylation/signaling	Potentially restrain abnormal VSMC proliferation, migration, and ECM remodeling when PDGFRβ is overactivated; not suitable for broad routine inhibition	Drug repositioning hypothesis requiring lesion stratification and safety validation
TGFβ pathway modulators	TGFβ/Smad and non-Smad branches	May regulate inflammation, fibrosis, EndMT, calcification, and plaque stability; effect depends on cell type and lesion stage	Experimental/translational exploration; systemic inhibition carries risk
LRP1-targeted nano-delivery	LRP1-mediated endocytosis	May deliver lipid-lowering, anti-inflammatory, miRNA, or PDGFRβ/TGFβ-modulating cargos to plaque cells; AS-specific validation is still needed	Potential precision-delivery strategy

## Data Availability

No new data were created or analyzed in this study. Data sharing is not applicable to this article.
